# Hypertension Is a Conditional Factor for the Development of Cardiac Hypertrophy in Type 2 Diabetic Mice

**DOI:** 10.1371/journal.pone.0085078

**Published:** 2014-01-09

**Authors:** Marc van Bilsen, Anneleen Daniels, Olaf Brouwers, Ben J. A. Janssen, Wouter J. A. Derks, Agnieszka E. Brouns, Chantal Munts, Casper G. Schalkwijk, Ger J. van der Vusse, Frans A. van Nieuwenhoven

**Affiliations:** 1 Department of Physiology, Cardiovascular Research Institute Maastricht, Maastricht University, Maastricht, the Netherlands; 2 Department of Internal Medicine, Cardiovascular Research Institute Maastricht, Maastricht University, Maastricht, the Netherlands; 3 Department of Pharmacology, Cardiovascular Research Institute Maastricht, Maastricht University, Maastricht, the Netherlands; 4 Department of Cardiology, Cardiovascular Research Institute Maastricht, Maastricht University, Maastricht, the Netherlands; Albert Einstein College of Medicine, United States of America

## Abstract

**Background:**

Type 2 diabetes is frequently associated with co-morbidities, including hypertension. Here we investigated if hypertension is a critical factor in myocardial remodeling and the development of cardiac dysfunction in type 2 diabetic db/db mice.

**Methods:**

Thereto, 14-wks-old male db/db mice and non-diabetic db/+ mice received vehicle or angiotensin II (AngII) for 4 wks to induce mild hypertension (n = 9–10 per group). Left ventricular (LV) function was assessed by serial echocardiography and during a dobutamine stress test. LV tissue was subjected to molecular and (immuno)histochemical analysis to assess effects on hypertrophy, fibrosis and inflammation.

**Results:**

Vehicle-treated diabetic mice neither displayed marked myocardial structural remodeling nor cardiac dysfunction. AngII-treatment did not affect body weight and fasting glucose levels, and induced a comparable increase in blood pressure in diabetic and control mice. Nonetheless, AngII-induced LV hypertrophy was significantly more pronounced in diabetic than in control mice as assessed by LV mass (increase +51% and +34%, respectively, p<0.01) and cardiomyocyte size (+53% and +31%, p<0.001). This was associated with enhanced LV mRNA expression of markers of hypertrophy and fibrosis and reduced activation of AMP-activated protein kinase (AMPK), while accumulation of Advanced Glycation End products (AGEs) and the expression levels of markers of inflammation were not altered. Moreover, AngII-treatment reduced LV fractional shortening and contractility in diabetic mice, but not in control mice.

**Conclusions:**

Collectively, the present findings indicate that type 2 diabetes in its early stage is not yet associated with adverse cardiac structural changes, but already renders the heart more susceptible to hypertension-induced hypertrophic remodeling.

## Introduction

Diabetes, hypertension, dyslipidemia and obesity are independent risk factors for the development of cardiovascular disease, with hypertension being the most common risk factor [1], [2]. Importantly, in diabetic patients a clustering of risk factors commonly occurs which markedly increases the risk for the development of cardiovascular pathology. Furthermore, both clinical and experimental studies suggested that diabetes by itself, i.e., in the absence of established hypertension or coronary artery disease, can already lead to abnormalities in cardiac function and structure. The latter condition is generally referred to as diabetic cardiomyopathy [3]–[5]. The exact cause and nature of the cardiac functional abnormalities in diabetic animals are still controversial. Much of the controversy might arise from the use of different animal models of, respectively, type 1 and type 2 diabetes and the age of the diabetic animals under study.

In two previous studies we found no evidence of marked cardiac dysfunction and structural remodeling in two animals models of type 2 diabetes, namely in adult db/db mice and in senescent Zucker Diabetic Fatty (ZDF) rats [6], [7]. This led us to conclude that type 2 diabetes *per se* does not lead to overt and clinically relevant cardiac dysfunction. Accordingly, we hypothesized that type 2 diabetes will lead to cardiomyopathy only in the presence of co-morbid factors such as hypertension. In the preclinical setting, so far the interaction between diabetes and hypertension has only been studied in various rat models of type 1 diabetes [8]-[11]. Accordingly, in the present study the interaction between type 2 diabetes and hypertension was studied in db/db mice by infusing a low dose of angiotensin II (AngII) to induce a relatively mild hypertension. Changes in left ventricular (LV) function and structural remodeling of the heart were determined. Advanced Glycation End products (AGEs) and the activation of AMP-activated protein kinase (AMPK) were assessed in heart tissue as putative mechanisms underlying cardiac remodelling. The collective findings indicate that in 18-wks-old db/db mice diabetes *per se* has limited structural and functional consequences for the heart. However, it does render the heart more susceptible to hypertension-induced hypertrophic remodelling.

## Methods

### Animal studies

All experiments were approved by the institutional animal ethics committee of the Maastricht University and performed according to European Union guidelines. During the entire experiment, mice had free access to standard chow (SNIFF, Soest, Germany) and drinking water and were housed in a temperature-controlled room with 12h:12h light-dark cycle. The mice were individually housed to prevent infliction of injuries. The animals were health checked by independent animal technicians on a daily basis.

Thirteen wks old male diabetic db/db (DM) mice and non-diabetic db/+ control (Cn) mice were obtained from Charles River, Calco, Italy (strain from Jackson mice: BKS.CG-M +/+ LEPR DB/JAX). At 14 wks of age, the mice were randomly allocated to four different groups: two AngII-treated groups (DM+Ang: n  =  11, Cn+Ang: n  =  11) and two vehicle-treated groups (DM: n  =  10, Cn: n  =  9). AngII was dissolved in sterile PBS and administered via subcutaneously implanted osmotic minipumps (ALZET, model 1004, DURECT Corporation, Cupertino, CA, USA) at a delivery dose of 1 mg/kg per day. Systolic and diastolic blood pressure in conscious mice was determined via the tail-cuff method using a Volume Pressure Recording sensor (CODA, KENT Scientific Corporation, Torrington, CT, USA) just before and 2 and 4 wks after the onset of treatment [12]. Additionally, at these time points blood glucose levels were determined in blood samples collected from the saphenous vein using the Contour glucosemeter (Bayer, Dublin, Ireland). Body weights were monitored weekly from 14 to 18 wks of age. Two animals in each of the groups treated with AngII (DM+Ang and Cn+Ang) died during the four week experimental period. Finally, at 18 wks of age anesthetized mice were subjected to a dobutamine stress test for determination of LV function. Thereafter, the animals were sacrificed and organs harvested for further analysis.

### Echocardiography

Cardiac dimensions and function were determined with echocardiography (Vevo 770 imaging system, Visualsonics, Toronto, Canada) under isoflurane-anesthesia (±2%) at 14, 16 and 18 wks of age, as previously described [7]. Wall thickness, and internal diameter, length and area of the LV cavity were assessed during diastole and systole. Additionally, heart rate (HR) was measured. From these parameters, fractional shortening (FS), ejection fraction (EF), end-diastolic and end-systolic volume (EDV and ESV), stroke volume (SV) and cardiac output (CO) were calculated. Echo-Doppler was applied to determine transmitral peak flow rates during the early diastolic (E) and atrial (A) LV filling phase and to calculate the E/A ratio, a measure of diastolic function.

### Dobutamine-stress test

Cardiac LV contraction and relaxation were evaluated 4 wks after the onset of AngII- or vehicle-treatment (at 18 wks of age) under baseline conditions and upon dobutamine stimulation to challenge the heart with increased workload [7]. Briefly, mice were anesthetized with urethane (2.5 mg/g body weight, i.p.) and a transducer-tipped catheter (Micro-Tip 3F, Millar Instruments, Houston, TX, USA) was inserted via the right carotid artery into the LV cavity. Maximal positive and negative pressure development (+/–dP/dt_max_) were determined at baseline and during stimulation with increasing amounts of dobutamine (0, 90, 180, 270, 360, 450, and 540 ng/min; 2 min per interval) infused via a second catheter inserted in the jugular vein.

### Immunohistochemistry, immunoblotting and enzyme activity

Immediately after the dobutamine-stress test, the anesthetized mice were sacrificed by exsanguination via the abdominal aorta. Heart, lungs, liver, and kidneys were harvested, weighed and processed for further analysis. Tibia length was measured with a marking gauge. The isolated heart was divided into atria, right ventricle (RV) and LV. Part of LV was fixed in buffered 4% formaldehyde for 24 h and imbedded in paraffin for histological evaluation of cardiomyocyte cross-sectional surface area and interstitial fibrosis. For this, tissue sections of 5 µm were fixed at 56°C overnight, deparaffinized, rehydrated and stained with hematoxylin and eosin (H&E) to determine cardiomyocyte cross-sectional surface area. To visualize interstitial fibrosis, the sections were stained with Picro-Sirius Red. The percentage of the LV wall consisting of interstitial collagen was calculated as the ratio of Picro-Sirius-Red positively stained area over total LV tissue area, excluding blood vessels. [7]. For CD45 staining slides were blocked in buffer containing normal Rabbit serum (Dako, Glostrup, Denmark), incubated with Anti-Mouse CD45 (BD Pharmingen, Franklin Lakes, NJ, USA) and then with biotin-conjugated secondary Rabbit anti-Rat antibody (Dako). Signal was amplified using TSA kit (PerkinElmer, Waltham, MA, USA) and visualized using DAB (Sigma, St. Louis, MO, USA).

For western blotting, LV tissue was homogenized in ice-cold lysis buffer containing phosphatase and protease inhibitors [7]. To determine the activation state of AMPK protein and glyoxylase-1 (GLO-1) protein level samples were separated by electrophoresis, blotted, and incubated with antibodies against Thr172 phosphorylated AMPK (Cell Signaling Technology, Danvers, MA, USA), GLO-1, and GAPDH for normalization. Polyclonal GLO-1 antibodies were raised in rabbits using a synthetic peptide based on human GLO-1.

GLO-1 activity was measured spectrophotometrically by the increase in absorbance (240 nm) due to formation of S*-D*-lactoyl-glutathione as described before [13].

### Protein-bound AGEs

The AGE adducts *N^ε^*-carboxymethyl-lysine (CML), *N^ε^*-carboxyethyl-lysine (CEL), and Methylglyoxal-derived-hydroxyimidazalone-1 (N^δ^-[5-hydro-5-methyl-4-imidazolon-2-yl]-L-ornithine, MG-H1) were measured in LV tissue homogenates with ultra-performance liquid chromatography tandem mass spectrometry (UPLC-MSMS) as described in detail elsewhere [14] and normalized to lysine residue concentration.

### Gene expression

Total RNA was isolated from LV tissue [7]. RNA purity and concentration were measured by means of the 260/280 nm ratio, and cDNA synthesis was performed using 250 ng RNA (Iscript cDNA synthesis kit, Biorad, Hercules, CA, USA). Gene expression was analyzed on an iCycler Real-Time PCR detection system using the iQ SYBR-green supermix (Biorad). Primers used are shown in Table S1. Results were normalized to the housekeeping gene Cyclophilin-A and relative changes in expression levels were calculated using the qBase analyzer.

### Statistics

Data are presented as mean±SEM and analyzed by one-way or two-way ANOVA with Bonferroni post-hoc testing when appropriate. Differences were considered statistically significant at a P-value <0.05.

## Results

### General characteristics

**Figure 1:**
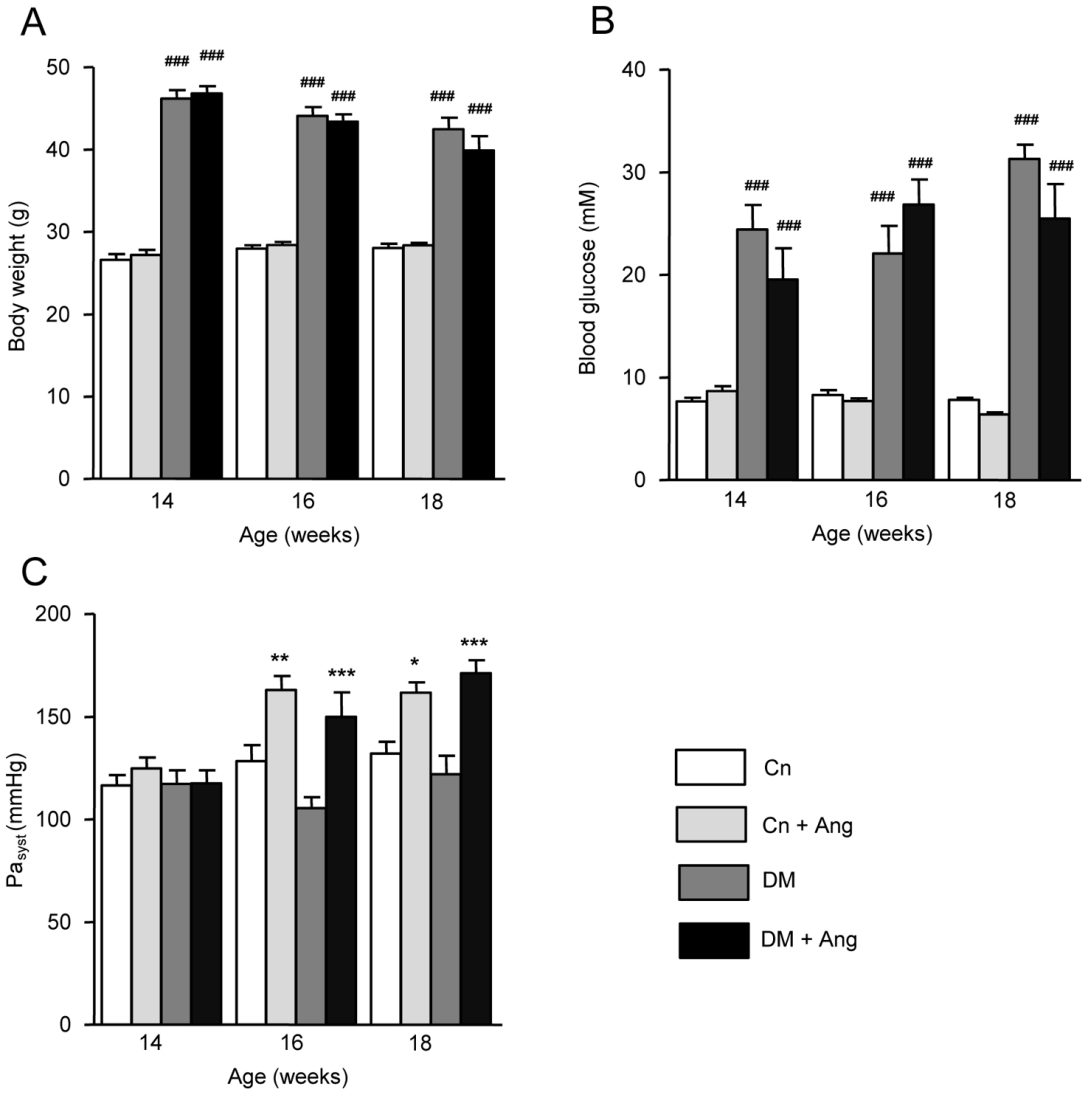
Effect of AngII treatment on blood pressure in non-diabetic and diabetic mice. Body weight (A), blood glucose levels (B) and systolic arterial blood pressure (Pa_syst_; C) of non-diabetic (Cn) and diabetic (DM) mice treated with vehicle or AngII (Ang) just prior to treatment (age 14 weeks) and at age 16 and 18 weeks. Data are analyzed by one-way ANOVA with Bonferroni post-hoc testing and expressed as mean±SEM (n  =  7–11 per group). * refers to effect of AngII in non-diabetic and diabetic mice (* P<0.05, ** P<0.01, *** P<0.001) and # refers to effect of diabetes in vehicle-treated and AngII-treated mice (# P<0.05, ## P<0.01, ### P<0.001).

The diabetic db/db mice (DM) demonstrated significantly higher body weights and fasting blood glucose levels when compared with the non-diabetic, control (Cn) mice from the start (14 wks) till the end of the experimental period (18 wks) (Figure 1A and 1B). Compared to the Cn mice, tibia length was slightly less (–6.5%, p<0.001) and liver weight was substantially higher (+ 50.2%, p<0.001) in DM mice (Table 1). Lung weight was not increased by diabetes or AngII-treatment (Table 1), indicating that pulmonary congestion was absent. Four wks of AngII-treatment did not affect body weight, tibia length, liver weight (Table 1) and blood glucose levels (Figure 1B), indicating that AngII did not influence obesity or the severity of diabetes.

**Table 1:** Morphometric characteristics of non-diabetic (Cn) and diabetic (DM) mice treated with vehicle or AngII (Ang) for 4 weeks.

	Cn	Cn+Ang	DM	DM+Ang
**Body Weight (g)**	28.1±0.3	28.6±0.4	42.4±1.4^###^	39.9±1.7^###^
**Tibia Length (mm)**	17.9±0.1	18.0±0.1	16.8±0.1^###^	16.8±0.2^###^
**Lung (mg)**	162±6	175±5	150±7	156±5
**Liver (mg)**	1329±58	1350±52	1997±85^###^	2066±147^###^
**Kidney (mg)**	192±5	192±4	209±5	188±8*
**Heart (mg)**	135±3	166±3***	128±3	182±5***^,#^
*Atria (mg)*	9.6±0.9	11.3±0.7	9.4±0.6	12.8±0.8*
*RV (mg)*	23±1.1	22±1.6	22±1.0	24±1.1
*LV (mg)*	94±4	126±4***	94±3	142±3***^,##^

Data are expressed as mean±SEM. RV, Right Ventricle; LV, Left Ventricle.

refers to effect of AngII in non-diabetic and diabetic mice (* P<0.05, ** P<0.01, *** P<0.001) and ^#^ refers to effect of diabetes in vehicle-treated and AngII-treated mice (# P<0.05, ## P<0.01, ### P<0.001).

### Cardiac function

Prior to AngII infusion blood pressure was similar in DM and Cn mice (Figure 1C). AngII-treatment increased blood pressure to the same extent in Cn+Ang and DM+Ang groups, both at 16 and 18 weeks of age. Before AngII-treatment, echocardiographically evaluated LV function did not differ among the experimental groups (Table S2). AngII-treatment for 4 wks did not affect LV function in non-diabetic mice: heart rate, ejection fraction (EF), fractional shortening (FS), end-diastolic and end-systolic volume, stroke volume and cardiac output did not change when compared to vehicle-treated mice (Table 2). The mitral E/A ratio, a measure of diastolic function, was comparable among the 4 groups investigated.

**Table 2:** Echo pulse-doppler determination of changes in cardiac dimensions and cardiac performance in non-diabetic (Cn) and diabetic (DM) mice treated with vehicle or angiotensin II (Ang) for 4 weeks.

	Cn	Cn+Ang	DM	DM+Ang
**Heart rate (beats/min)**	357±19	381±24	385±19	429±17
**Wall thickness, diastolic (mm)**	0.71±0.02	0.84±0.03	0.77±0.02	0.96±0.06**
**Wall thickness, systolic (mm)**	1.14±0.03	1.31±0.03	1.30±0.05	1.46±0.08
**End-diastolic volume, long-axis (µl)**	55±2	64±3	49±5	58±3
**End-systolic volume, long-axis (µl)**	21±3	26±2	16±2	23±2*
**Ejection fraction (%)**	63±3	60±2	69±3	61±3
**Fractional shortening (%)**	28.0±2.1	27.1±0.7	37.6±2.4^#^	28.6±3.3*
**Stroke volume (µl)**	34±2	38±2	33±3	36±3
**Cardiac output (ml.min^−1^)**	12.4±1.1	14.7±1.4	12.8±1.3	15.1±1.1
**E/A ratio**	1.69±0.17	1.75±0.13	2.10±0.14	1.76±0.20

Data are expressed as means ± S.E.M. * refers to effect of angiotensin II in non-diabetic and diabetic mice (* P<0.05, ** P<0.01, *** P<0.001) and ^#^ refers to effect of diabetes in vehicle-treated and Angiotensin II-treated mice (# P<0.05, ## P<0.01, ### P<0.001).

**Figure 2:**
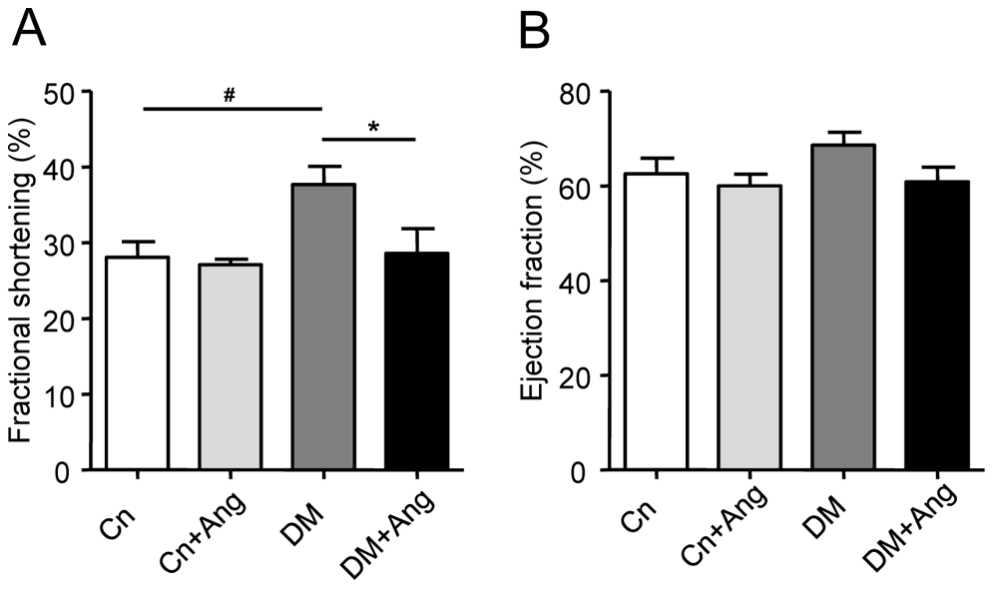
Effect of AngII-induced hypertension on cardiac function in non-diabetic and diabetic mice. Left ventricular fractional shortening (A) and ejection fraction (B) as determined by echocardiography in non-diabetic (Cn) and diabetic (DM) mice treated with vehicle or AngII (Ang) for 4 weeks (age 18 weeks). Data are analyzed by one-way ANOVA with Bonferroni post-hoc testing and expressed as mean±SEM (n  =  7–9 per group). * refers to effect of AngII in non-diabetic and diabetic mice (* P<0.05) and ^#^ refers to effect of diabetes in vehicle-treated and AngII-treated mice (# P<0.05, ## P<0.01, ### P<0.001).

At 18 weeks of age, FS was higher in vehicle-treated diabetic (DM) mice than in non-diabetic Cn mice (Figure 2A). AngII-treatment did not affect FS in non-diabetic mice. However, FS was significantly lower in the DM+Ang group than in the vehicle-treated DM group. Differences in EF were not observed (Figure 2B).

**Figure 3:**
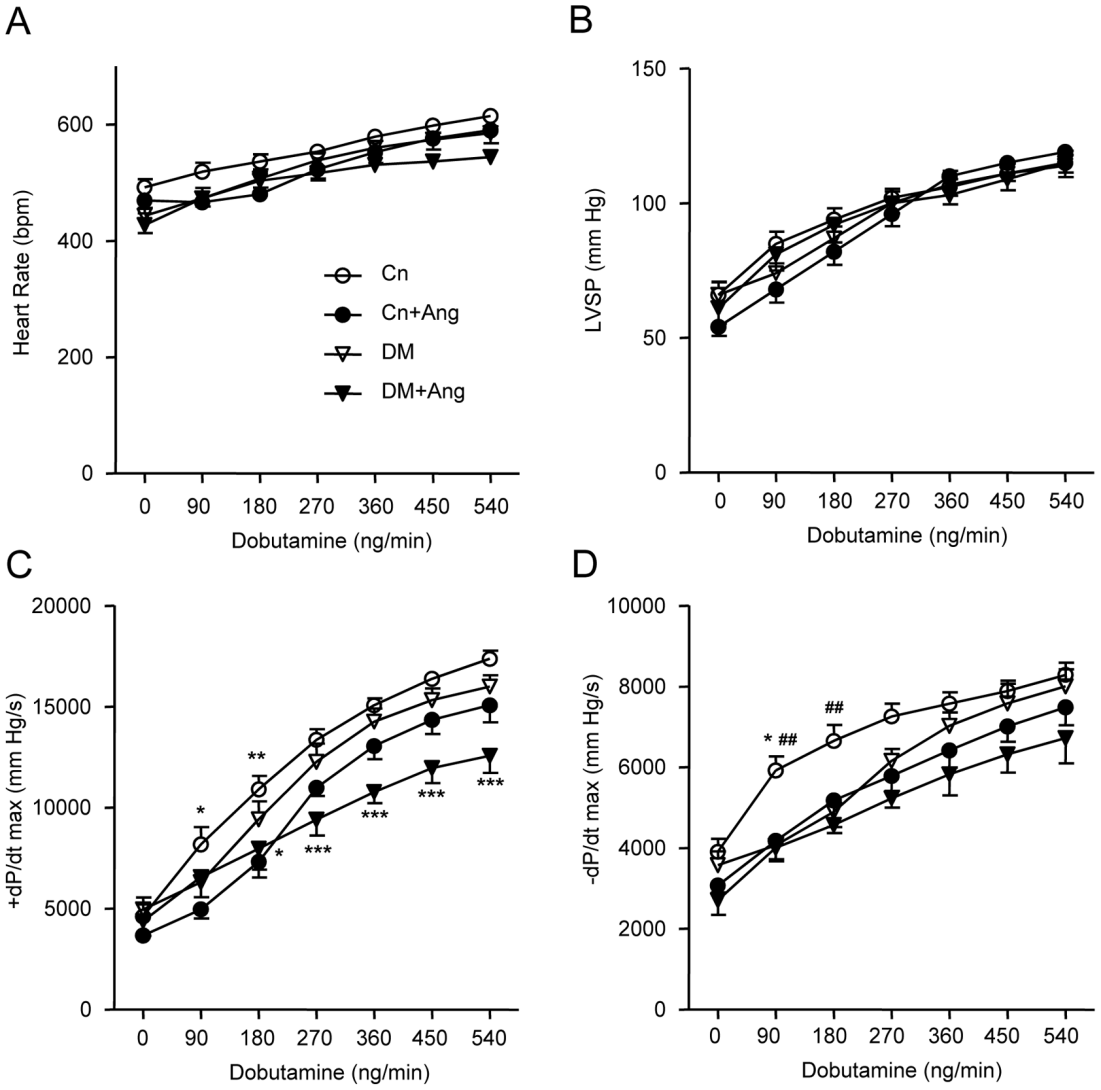
Effect of AngII-induced hypertension on left ventricular function during dobutamine stimulation. Hemodynamic analysis of left ventricular function in non-diabetic (Cn) and diabetic (DM) mice treated with vehicle or AngII (Ang) for 4 weeks (age 18 weeks), at baseline and during dobutamine stimulation. A) heart rate, B) peak systolic left ventricular pressure (LVSP), C) maximal positive pressure development (+dP/dt_max_) and D) maximal negative pressure development (–dP/dt_max_). Data are analyzed by two-way ANOVA with Bonferroni post-hoc testing and expressed as mean±SEM (n  =  7–9 per group). * refers to effect of AngII in non-diabetic and diabetic mice (* P<0.05, ** P<0.01, *** P<0.001) and ^#^ refers to effect of diabetes in vehicle-treated and AngII-treated mice (# P<0.05, ## P<0.01, ### P<0.001).

During the dobutamine-stress test significant differences in heart rate (HR), LV systolic pressure (LVSP), and positive and negative dP/dt_max_ were observed at baseline (Figure 3A-D). When the mice were exposed to step-wise enhanced concentrations of dobutamine, HR and LVSP increased to the same extent in the 4 experimental groups (Figure 3A and 3B). However, the dobutamine-induced increase of +dP/dt_max_ was blunted in AngII-treated diabetic mice compared to vehicle-treated DM mice (Figure 3C). The LV relaxation parameter -dP/dt_max_ showed a statistically smaller increase under dobutamine-stimulation at low doses in both DM and AngII-treated non-diabetic mice compared to Cn animals, indicating similar effects of diabetes and hypertension on relaxation velocity. However, both at baseline and at high doses of dobutamine, no differences in –dP/dt_max_ were observed between the four groups (Figure 3D).

### Cardiac remodeling

**Figure 4:**
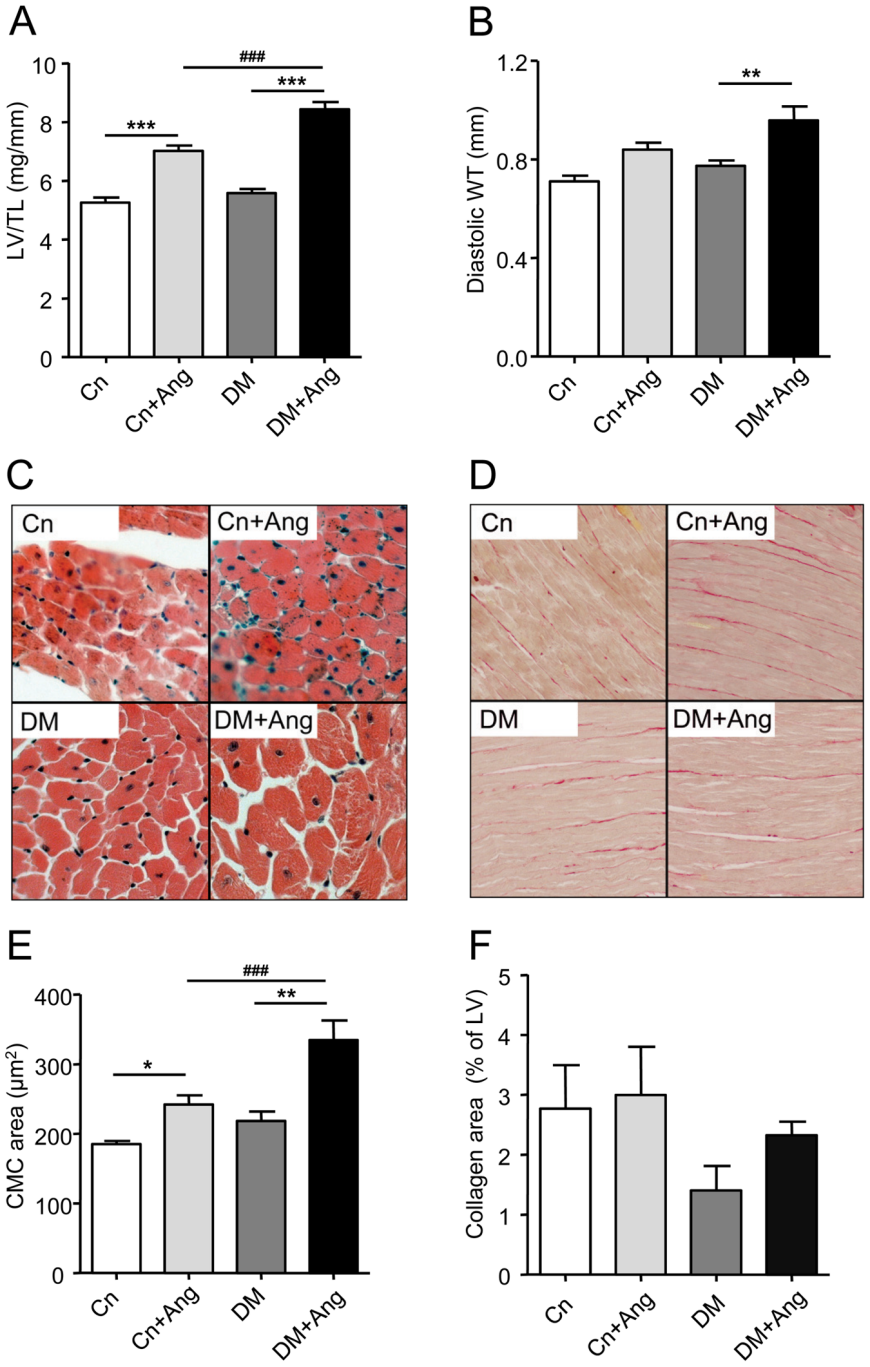
AngII-induced hypertension enhances cardiac hypertrophy in diabetic mice. Cardiac structural remodeling in non-diabetic (Cn) and diabetic (DM) mice treated with vehicle or AngII (Ang) for 4 weeks. Left ventricle weight/Tibia length (LV/TL – Panel A) and diastolic LV wall thickness (WT – Panel B). Representative pictures depicting cardiomyocyte size (C) and extent of fibrosis (D). Quantification of cardiomyocyte cross-sectional surface area (E) and collagen fraction (F). Data are analyzed by one-way ANOVA with Bonferroni post-hoc testing and expressed as mean±SEM (n  =  7–9 per group). * refers to effect of AngII in non-diabetic and diabetic mice (* P<0.05, ** P<0.01, *** P<0.001) and ^#^ refers to effect of diabetes in vehicle-treated and AngII-treated mice (# P<0.05, ## P<0.01, ### P<0.001).

Total heart and LV weight were similar in the vehicle-treated DM and the non-diabetic Cn mice (Table 1). AngII-treatment increased total heart and LV weight in both groups, the increase being more pronounced in de diabetic mice than in non-diabetic mice (Table 1). LV/TL was significantly increased in AngII-treated animals, and LV/TL is significantly higher in DM+Ang compared to Cn+Ang (Figure 4A). Echocardiographic analysis revealed that diastolic wall thickness was significantly increased in AngII-treated diabetic mice 2 and 4 wks after initiation of AngII-treatment (Table 2, Table S2, and Figure 4B). In Cn+Ang mice the increase in diastolic wall thickness did not reach the level of statistical significance. Furthermore, at the level of the cardiomyocyte the AngII-mediated increase in cardiomyocyte cross sectional area was more outspoken in the diabetic mice than in the non-diabetic mice (+52% and +31% for DM+Ang and Cn+Ang mice, respectively) (Figure 4C, 4E). In line with this AngII-treatment significantly increased the mRNA expression of the hypertrophy markers brain natriuretic peptide (BNP) and alpha-skeletal actin (αSKA) in DM+Ang mice only (Table 3).

**Table 3:** Left ventricular mRNA levels of genes signifying changes in cardiac hypertrophy, metabolism, fibrosis, inflammation and AGE signalling in non-diabetic (Cn) and diabetic (DM) mice treated with vehicle or AngII (Ang) for 4 weeks.

	Cn	Cn+Ang	DM	DM+Ang
**Hypertrophy**				
ANP	1.0±0.1	2.3±0.6	0.6±0.1	2.3±0.6
BNP	1.0±0.1	1.4±0.2	0.3±0.1^###^	0.9±0.3*^,#^
αSKA	1.0±0.1	4.9±0.9	1.0±0.7	8.4±2.2***
**Metabolism**				
AngPtl4	1.0±0.2	1.2±0.3	2.9±1.2	3.2±0.9
UCP3	1.0±0.2	0.7±0.2	2.1±0.6	1.6±0.4
**Fibrosis**				
αSMA	1.0±0.1	1.1±0.1	1.4±0.5	1.7±0.4
Col1	1.0±0.1	2.0±0.2	1.4±0.3	4.7±0.9***^,##^
Col3	1.0±0.0	1.7±0.2	0.9±0.2	3.6±0.7***^,##^
Col4	1.0±0.1	1.2±0.1	1.4±0.4	2.8±0.7^#^
CTGF	1.0±0.1	1.9±0.1	0.9±0.1	2.5±0.5***
MMP2	1.0±0.1	1.6±0.1	1.2±0.2	1.9±0.2*
MMP9	1.0±0.2	0.6±0.1	0.8±0.2	0.8±0.2
**Inflammation**				
IκBα	1.0±0.1	0.9±0.1	1.2±0.2	0.9±0.1
IL-6	1.0±0.1	0.9±0.2	0.3±0.1^##^	0.7±0.2
**AGE’s**				
GLO-1	1.0±0.1	1.0±0.1	1.2±0.1	1.1±0.1
RAGE	1.0±0.1	1.0±0.1	1.1±0.1	1.2±0.1
AGER-1	1.0±0.0	0.9±0.0	1.0±0.0	0.9±0.0

Data are expressed relative to non-diabetic vehicle-treated mice as mean±SEM. Abbreviations are explained in Table S1. * refers to effect of AngII in non-diabetic and diabetic mice (* P<0.05, ** P<0.01, *** P<0.001) and ^#^ refers to effect of diabetes in vehicle-treated and AngII-treated mice (# P<0.05, ## P<0.01, ### P<0.001).

Histological analysis revealed no significant effect of diabetes and AngII, either alone or in combination, on LV collagen content (Figure 4D, 4F). The LV expression of fibrotic marker genes, i.e. alpha-smooth muscle actin (αSMA), type I collagen (col1), type III collagen (col3), type IV collagen (col4), connective tissue growth factor (CTGF), matrix metalloproteinase-2 (MMP2) and matrix metalloproteinase-9 (MMP9), was comparable between DM and Cn mice. AngII-treatment increased the expression of most fibrotic genes, but the increase only reached statistical significance in the DM+Ang group (Table 3).

### Cardiac inflammation

As diabetes is associated with a state of chronic low-grade inflammation [15], [16]we explored if differences in inflammation might be responsible for the increased hypertrophic remodeling in the AngII-treated diabetic mice. In the myocardium of Cn mice the number of CD45-positive cells amounted to 49±10 cells/mm^2^. The number of leukocytes significantly increased after AngII-treatment of non-diabetic mice (90±13 cells/mm^2^, p<0.05), but remained unchanged in non-treated (DM) and AngII-treated diabetic (DM+Ang) mice. mRNA expression of the inflammatory marker nuclear factor-kappa B inhibitor-alpha (IκBα) was not affected, neither by diabetes nor by AngII-treatment (Table 3). Interleukin-6 (IL-6) expression was even significantly lower in vehicle-treated DM mice than in their non-diabetic counterparts. Taken together, Ang-II treatment did not affect the expression of the inflammatory marker studied.

### Advanced Glycation End products (AGEs)

**Figure 5:**
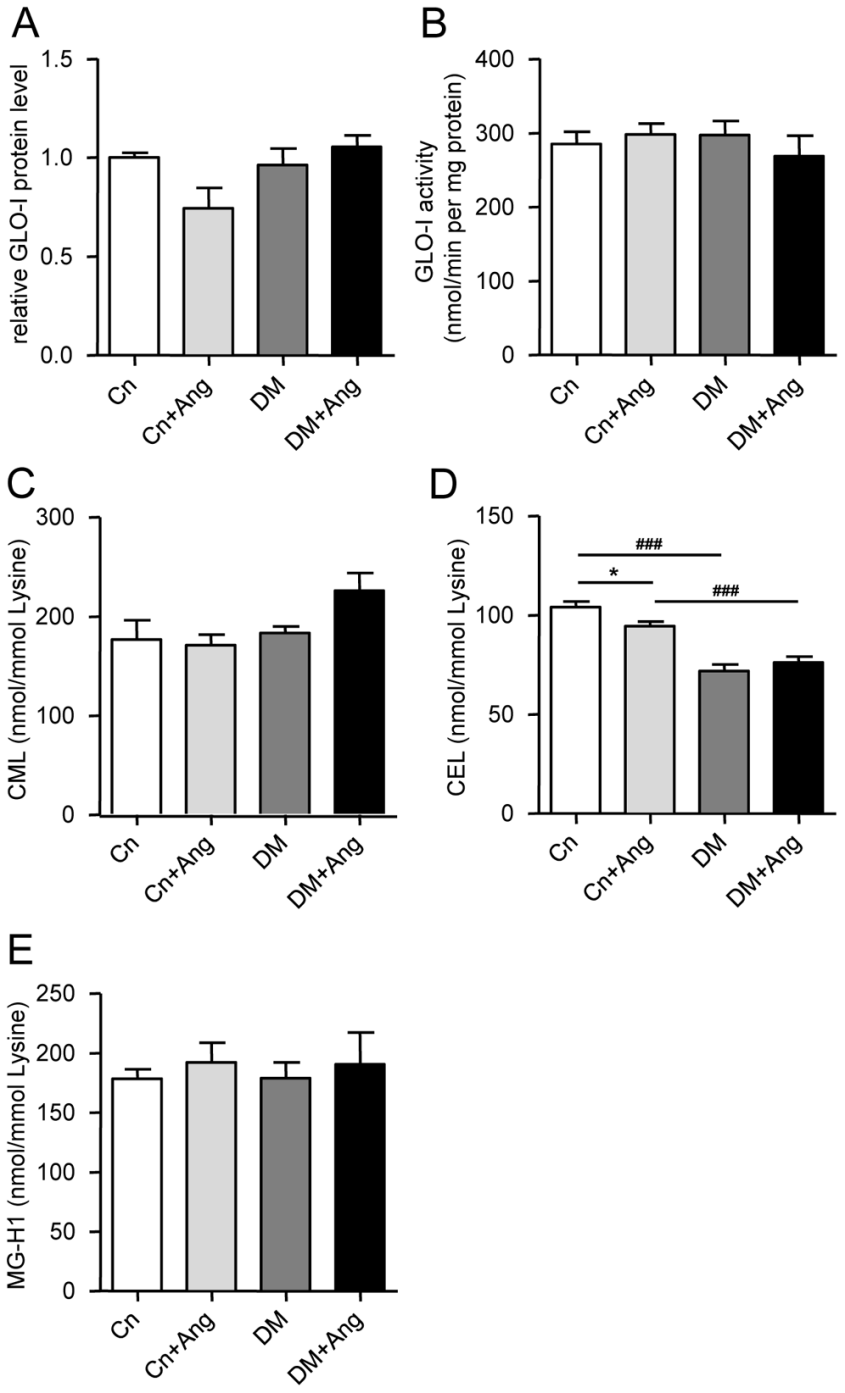
Effect of hypertension on cardiac AGE metabolism in non-diabetic and diabetic mice. Cardiac levels of GLO-1 protein (A), GLO-1 activity (B), CML (C), CEL (D), and MG-H1 (E) in non-diabetic (Cn) and diabetic (DM) mice treated with vehicle or AngII (Ang) for 4 weeks (age 18 weeks). Data are analyzed by one-way ANOVA with Bonferroni post-hoc testing and expressed as mean±SEM (n  =  4–6 per group) * refers to effect of AngII in non-diabetic and diabetic mice (* P<0.05, ** P<0.01, *** P<0.001) and ^#^ refers to effect of diabetes in vehicle-treated and AngII-treated mice (# P<0.05, ## P<0.01, ### P<0.001).

AGEs are considered to play a central role in cardiovascular pathology in diabetes [17]. Hence, we explored if accumulation of AGEs in the myocardium could account for the increased hypertrophy in AngII-treated diabetic mice. Neither diabetes nor AngII affected the mRNA level (Table 3), protein level (Figure 5A) or the catalytic activity (Figure 5B) of GLO-1, the enzyme responsible for the detoxification of the major AGE-precursor methylglyoxal, to a significant extent. The mRNA levels of the Receptor for AGEs (RAGE), the activation of which leads to NFκB signaling and hypertrophy, and of the AGE Receptor-1 (AGER-1, also referred to as dolichyl-diphosphooligosaccharide-protein glycosyltransferase), acting as a scavenging receptor for AGEs, did not change either (Table 3).

Next we measured the LV tissue content of various AGEs by UPLC-MSMS (Figure 5C - E). Myocardial levels of the arginine adduct MG-H1 were not affected by diabetes, AngII-treatment, or the combination of both. Surprisingly, tissue levels of the lysine adduct CEL were markedly lower in both vehicle-treated and AngII-treated db/db mice, whereas tissue levels of CML, another lysine adduct, did not change.

### Cardiac metabolism

**Figure 6:**
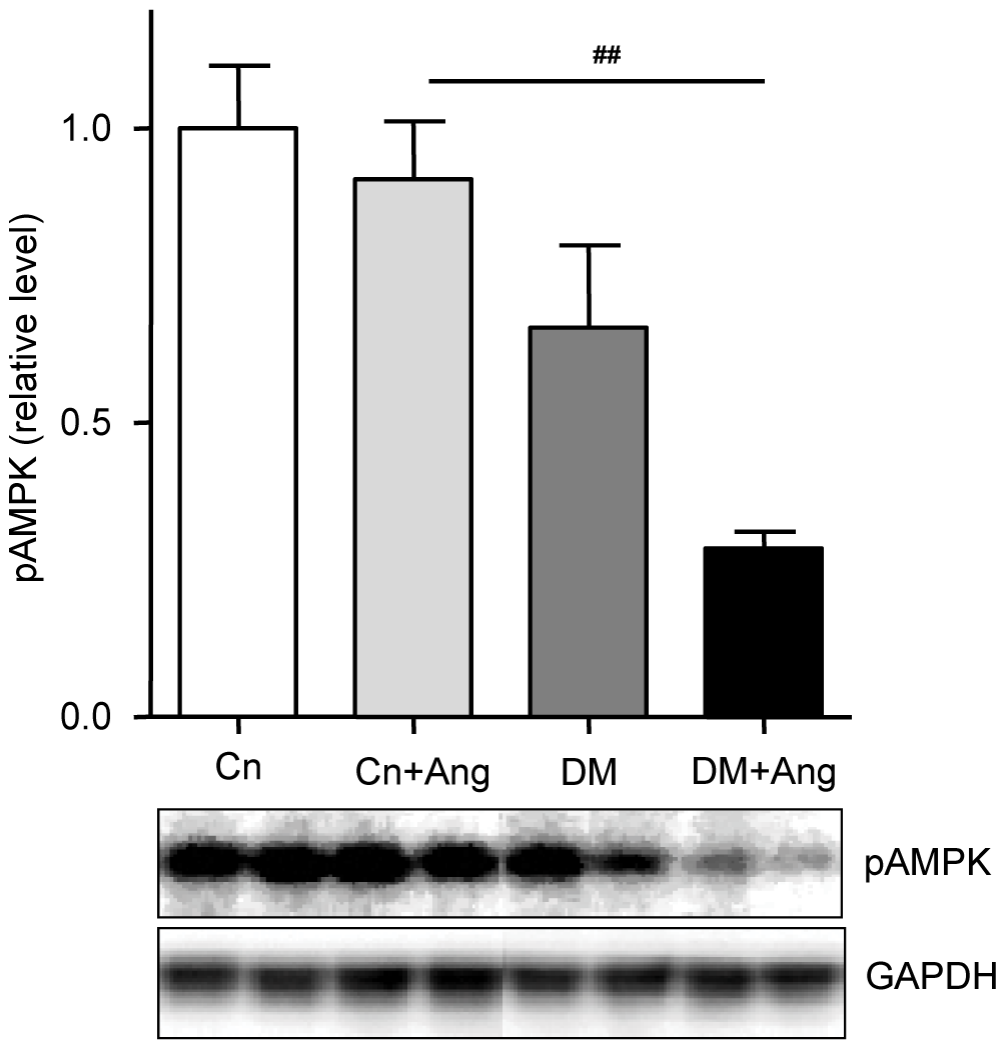
Cardiac phospho-AMPK levels are decreased in hypertensive, diabetic mice. Western blot analysis of LV pAMPK levels in non-diabetic (Cn) and diabetic (DM) mice treated with vehicle or AngII (Ang) for 4 weeks. In the top panel, the quantification of LV pAMPK levels from 4–6 animals per group is shown. Data are analyzed by one-way ANOVA with Bonferroni post-hoc testing and expressed as mean±SEM. In the bottom panel, representative examples of pAMPK and GAPDH signals are shown for 2 animals per group. * refers to effect of AngII in non-diabetic and diabetic mice (* P<0.05, ** P<0.01, *** P<0.001) and # refers to effect of diabetes in vehicle-treated and AngII-treated mice (# P<0.05, ## P<0.01, ### P<0.001).

Finally, we investigated if changes related to cardiac metabolism might contribute to the increased hypertension-induced hypertrophic remodelling in the diabetic mice. Compared to corresponding non-diabetic controls and consistent with the diabetic phenotype, the expression of metabolic marker genes angiopoietin-like 4 (Angptl4) and uncoupling protein 3 (UCP3) tended to be higher in both vehicle-treated and AngII-treated db/db mice (Table 3). However, due to the large inter-individual variation these differences didn’t reach the level of significance. Consistent with our previous findings [7] cardiac protein levels of pAMPK tended to be lower in vehicle-treated diabetic mice than in non-diabetic controls (Figure 6; p = 0.08). Interestingly, AngII-treatment had little effect in non-diabetic controls, but was associated with a profound decline (decrease 53%) in cardiac pAMPK levels in diabetic mice.

## Discussion

In the present study the interaction between type 2 diabetes and hypertension was investigated in 14–18 wks old db/db mice as an animal model of type 2 diabetes using AngII infusion to induce hypertension. The main finding is that type 2 diabetes *per se* is not a strong trigger for structural remodeling and cardiac dysfunction, but that cardiac hypertrophy is more pronounced subsequent to a chronic, moderate increase in blood pressure. We conclude that the diabetic heart is more susceptible to hypertrophic remodelling in the presence of hypertension.

To the best of our knowledge this is the first study investigating the interaction between type 2 diabetes and hypertension on cardiac function and structure. Previous studies concentrated on the effect of type 1 diabetes and hypertension. Despite highly diverse results, these studies show that hypertension and type 1 diabetes have independent, at the best additive effects, on hypertrophy, cardiac dysfunction, or fibrosis [8]–[11]. For instance, Falcao-Pires and coworkers, using suprarenal aortic banding to induce hypertension and streptozotocin to induce type 1 diabetes in rats, concluded that diabetes was largely responsible for increasing LV stiffness, whereas hypertension led to impaired cardiomyocyte relaxation, thereby both contributing to the diastolic dysfunction [9]. In their study the increase in LV mass was caused exclusively by the pressure overload, while diabetes had no additive effect. This is in striking contrast to the present study showing that indeed type 2 diabetes by itself has no effect, but that combined with AngII-induced hypertension diabetes appears to potentiate hypertrophic remodelling.

### Diabetes and cardiac function

Db/db mice herald several features of human type 2 diabetes, combining obesity, dyslipidemia, insulin resistance, hyperglycemia and albuminuria [7], [18], [19]. In line with a previous study, the presence of severe diabetes in normotensive db/db mice only leads to mild cardiac dysfunction and virtually no signs of structural remodeling [7]. In fact, left ventricular fractional shorting (FS) was even somewhat higher in the normotensive diabetic mice. Other studies also reported improved [20] or preserved [19] systolic function in db/db mice of comparable age. The increased function in db/db mice has been attributed to favourable changes in load-dependent [21] as well as load-independent factors [22]. More recently we showed that even in aged ZDF fatty rats suffering from long-lasting and severe diabetes, cardiac function was largely preserved, fibrosis was only slightly increased, and cardiomyocyte hypertrophy was absent [6]. These collective findings suggest that type 2 diabetes *per se* does not result in overt cardiac remodeling and dysfunction. This notion led us to postulate that the diabetic condition rather sensitizes the heart to other common co-morbid factors, like hypertension. Accordingly, in this study hypertension was installed by continuous infusion of a relatively low dose of AngII, resulting in a 30–40 mmHg rise in blood pressure. Notably, the AngII-induced rise in blood pressure was comparable between diabetic db/db and non-diabetic db/+ mice, thereby excluding that differences in blood pressure account for the observed differences in cardiac hypertrophy.

### Cardiac function and remodeling in hypertensive db/db mice

In the non-diabetic mice the AngII-induced mild hypertension did not alter any of the echocardiographically determined LV functional parameters. In addition, cardiac function in the AngII-treated db/db mice was still largely maintained, the only differences being a lower FS compared to the non-treated db/d/b mice and a reduced response of the +dp/dt_max_ in the dobutamine stress test, which may be interpreted of signs of mild systolic dysfunction. Indices of diastolic function (E/A ratio; –dp/dt_max_) in vehicle- and AngII-treated DM mice were not significantly affected at baseline, or at maximal dose of dobutamine stimulation. This is in line with the absence of an increase in collagen staining in the diabetic hearts, which precludes a role of extracellular matrix (ECM) deposition as a determinant of cardiac diastolic function in these mice. It should be noted, however, that mRNA expression of collagens I, III and IV was increased to a larger extent in the AngII-treated db/db mice than in the AngII-treated non-diabetic mice. Moreover, CTGF and MMP2 expression was also elevated, suggesting that active ECM remodeling is taking place [23], without resulting in significantly increased deposition of ECM during the study period. It remains open to discussion if after longer treatment with AngII the observed increase in the expression of ECM components would have resulted in increased ECM deposition and, eventually, cardiac diastolic dysfunction.

Using several independent approaches to assess hypertrophic growth (LV weight, echocardiography, histology) we demonstrated that type 2 diabetes by itself has no effect. However, type 2 diabetes renders the heart more susceptible to hypertrophic growth induced by AngII-induced hypertension. At present it is incompletely understood what causes the increased sensitivity of the diabetic heart to develop hypertension-induced hypertrophy.

### Mechanism of diabetes-induced increased sensitivity to hypertrophic remodeling

As db/db mice are still severely hyperinsulinemic at the age of 18 wks [7], [19], it is tempting to speculate that the enhanced sensitivity for hypertrophic remodeling is caused by the growth-stimulating properties of insulin. However, hyperinsulinemia is believed to be a compensatory response to insulin resistance, which is related to a defect in signaling downstream of the insulin-receptor in many organs, including the heart. Furthermore, the vehicle-treated db/db mice also suffer from hyperinsulinemia, but do not show evidence of cardiac hypertrophy. Hence, it is unlikely that insulin-mediated PI3-kinase/Akt signaling is responsible for the increased cardiac growth in the hypertensive diabetic mice.

Diabetes is considered a state of chronic subclinical inflammation and inflammatory signaling is believed to promote cardiac hypertrophy [15], [16], [24]. However, expression of the pro-inflammatory cytokine IL-6, and of IκBα, a marker of the activation state of the NFκB-pathway [25], was not increased in any of the groups. In fact, IL-6 mRNA levels were even reduced in DM mice. Moreover, following AngII treatment the number of infiltrating leucocytes (CD45^+^ cells) was increased in non-diabetic, but not in diabetic mice. These findings argue against a pivotal role of inflammatory signaling as being responsible for the pro-hypertrophic cardiac effect observed in hypertensive type 2 diabetic mice.

Enhanced formation of AGEs during diabetes is generally believed to play a pivotal role cardiac pathophysiology [17], [26]. Remarkably, despite the dramatically increased plasma glucose levels the myocardial content of protein-bound AGE products was either unaffected (MG-H1) or even declined (CEL) in the diabetic groups. The reduction in glucose uptake by insulin-resistant cardiomyocytes might explain the absence of an increase in AGEs in this organ. Only the tissue content of CML tended to increase in the hearts of AngII-treated diabetic mice. Interestingly, the latter AGE product can also be generated via lipid peroxidation, and therefore might signify enhanced lipid loading rather than glucose loading of the diabetic heart. As far as we know this is the first study documenting the concentration of AGEs, as measured with state-of the art mass spectrometry technology in myocardial tissue in a model of type 2 diabetes. It shows that the cardiac content of AGEs does not increase significantly as a result of diabetes and/or hypertension, which does not support an important role of intracellular AGEs in explaining the differences in cardiac hypertrophy as seen in the present study.

Finally, as it is well-established that activation of AMPK blunts the hypertrophic response [27], [28] we explored whether increased susceptibility for hypertrophic remodeling may relate to changes in cardiac metabolism, more specifically the activation state of cardiac AMPK. Indeed, only in diabetic AngII-treated mice a significant decline in pAMPK, reflecting reduced AMPK activation, was observed (see Figure 6). In line, recently an important role of AMPK in cardiac remodeling was demonstrated in leptin and LDL-receptor deficient mice, another model of the metabolic syndrome [29]. Diminished AMPK activation may therefore play a role in the increased sensitivity of the diabetic heart to developing hypertension-induced cardiac growth, as the brake to develop hypertrophy is less powerful.

### Limitations of the study

In the present study we investigated the interaction between type 2 diabetes and AngII-induced hypertension. The present experimental set-up, however, does not allow us to discern if the increased hypertrophic response of the diabetic heart reflects an increased sensitivity to hypertension or to AngII *per se*. There is controversy about the development of hypertension in db/db mice as some studies reported an increase [30], [31], while others reported no change in blood pressure [19], [32]. The hypertension observed by some investigators has been attributed to increased plasma AngII levels secondary to changes in angiotensin converting enzyme [31] and angiotensin converting enzyme-2 [33] activity in diabetic animals. In the present study blood pressure was not elevated in awake 14–18 wks old diabetic db/db mice as compared to age-matched non-diabetic db/+ controls. Also the rise in blood pressure after AngII administration was comparable in the db/+ and db/d/b mice. These observations make it less likely that differences in AngII processing are responsible for the increased hypertrophy in the diabetic mice.

Similar to hypertension, controversial findings have been reported on the presence of cardiac hypertrophy and fibrosis in db/db mice. This might be related to genetic background, sex, age and diet of the mice under study. Some studies reported fibrosis or hypertrophy already at 8–12 wks of age [21], [34] and others reported no changes [35], or only changes at much older age [36]. In this study we analysed 18-wks-old, male mice on a C57Bl/KSJ background (which develop a more severe phenotype than the C57Bl/6J background) and found no evidence of structural cardiac remodelling. As the complications of diabetes, including the effects on the heart, might worsen with time, it will be interesting to explore if older db/db mice do develop hypertrophy and fibrosis and if imposition of hypertension on top of that renders diabetic mice more vulnerable to the development of heart failure.

### Concluding remarks

Patients with diabetes have a higher risk of developing heart failure [37] and patients with acute heart disease have a poorer prognosis when also suffering from diabetes [38]. Diabetic patients are often characterized by a clustering of co-morbidities. The present observations in a mouse model of type 2 diabetes show that diabetes, in its initial stages, does not lead to marked structural remodeling of the heart. However, in combination with hypertension, a cardiovascular risk factor frequently associated with type 2 diabetes, the diabetic heart is more sensitive to hypertrophic remodeling than the non-diabetic heart. It is worthy to note that in patients referred for aortic valve replacement, cardiac fibrosis and diastolic dysfunction are more outspoken when they also suffer from diabetes [39]. The marked reduction in AMPK phosphorylation as seen in the hypertensive diabetic mice suggests that changes in cardiac AMPK activity may contribute to the increased vulnerability of the diabetic heart to develop hypertrophy, but further studies are warranted to elucidate the molecular mechanism in full detail.

## Supporting Information

Table S1 Gene-specific primer sequences used for quantitative real-time PCR.
(DOCX)

Table S2 Echo pulse-doppler determination of cardiac dimensions and cardiac performance as a function of time in non-diabetic (Cn) and diabetic (DM) mice treated with vehicle or angiotensin II (Ang) for 4 weeks.
(DOC)
