# Supplementary material for: Hypertension Is a Conditional Factor for the Development of Cardiac Hypertrophy in Type 2 Diabetic Mice
Source: PLoS One. 2014 Jan 9;9(1):e85078. doi: 10.1371/journal.pone.0085078 (PMC3887022; doi:10.1371/journal.pone.0085078)
Supplement: Table S1 — Gene-specific primer sequences used for quantitative real-time PCR. (DOCX) [file pone.0085078.s001.docx]

**Supplementary Table S1.** Gene-specific primer sequences used for quantitative real-time PCR

| **Gene** | **Forward-primer** | **Reversed-primer** |
| --- | --- | --- |
| α-skeletal actin (αSKA) | TGAGACCACCTACAACAGCA | CCAGAGCTGTGATCTCCTTC |
| α-smooth muscle actin (αSMA) | GTCCCAGACATCAGGGAGTAA | TCGGATACTTCAGCGTCAGGA |
| AGER-1 | CCCGGACAATCCCTTGGTTT | TGAGAATACCTCTGCGCACC |
| atrial natriuretic peptide (ANP) | ATTGACAGGATTGGAGCCCAGAGT | TGACACACCACAAGGGCTTAGGAT |
| angiopoietin-like 4 (AngPtl4) | CCTTTCCCTGCCCTTCTCTAC | AGGCTCTTGGCACAGTTAAGG |
| brain natriuretic peptide (BNP) | GCCAGTCTCCAGAGCAATTC | CCTTGGTCCTTCAAGAGCTG |
| type I collagen (Col1) | CGAAGGCAACAGTCGCTTCA | GGTCTTGGTGGTTTTGTATTCGAT |
| type III collagen (Col3) | TCGGAACTGCAGAGACCTAAA | CCCCAGTTTCCATGTTACAGA |
| type IV collagen (Col4) | TGTCATGGTGTGAAGGGACA | TCTCCAGCATCACCCTTTTG |
| connective tissue growth factor (CTGF) | CACAGAGTGGAGCGCCTGTTC | GATGCACTTTTTGCCCTTCTTAATG |
| cyclophilin-A (Cyclo) | CAAATGCTGGACCAAACACAA | TTCACCTTCCCAAAGACCACAT |
| Glyoxylase 1 (Glo-1) | ATGACGAGACTCAGAGTTACCACAA | TAGACATCAGGAACGGCAAATCC |
| IκBα | TGGAAGTCATTGGTCAGGTGAA | CAGAAGTGCCTCAGCAATTCCT |
| Interleukin-6 (IL-6) | CTGCAAGAGACTTCCATCCAGTT | GAAGTAGGGAAGGCCGTGG |
| matrix metalloproteinase 2 (MMP2) | TTTGCTCGGGCCTTAAAAGTAT | CCATCAAACGGGTATCCATCTC |
| matrix metalloproteinase 9 (MMP9) | CAAATTCTTCTGGCGTGTGA | CGGTTGAAGCAAAGAAGGAG |
| receptor for AGE’s (RAGE) | GCACTTAGATGGGAAACTTCTGATTC | GAGTCCCGTCTCAGGGTGTCT |
| uncoupling protein 3 (UCP3) | GGATTTGTGCCCTCCTTTCTG | CATTAAGGCCCTCTTCAGTTGCT |
