# Supplementary material for: Hypertension Is a Conditional Factor for the Development of Cardiac Hypertrophy in Type 2 Diabetic Mice
Source: PLoS One. 2014 Jan 9;9(1):e85078. doi: 10.1371/journal.pone.0085078 (PMC3887022; doi:10.1371/journal.pone.0085078)
Supplement: Table S2 — Echo pulse-doppler determination of cardiac dimensions and cardiac performance as a function of time in non-diabetic (Cn) and diabetic (DM) mice treated with vehicle or angiotensin II (Ang) for 4 weeks. (DOC) [file pone.0085078.s002.doc]

**Supplementary Table S2.** Echo pulse-doppler determination of cardiac dimensions and cardiac performance as a function of time in non-diabetic (Cn) and diabetic (DM) mice treated with vehicle or angiotensin II (Ang) for 4 weeks.

|  | **Cn** | **Cn+Ang** | **DM** | **DM+Ang** |
| --- | --- | --- | --- | --- |
| Heart rate (beats/min) |  |  |  |  |
| *- 14 wks* | 379±20 | 386±11 | 388±14 | 369±10 |
| *- 16 wks* | 403±19 | 401±13 | 404±12 | 428±10 |
| *- 18 wks* | 357±19 | 381±24 | 385±19 | 429±17 |
| *Wall thickness, diastolic (mm)* |  |  |  |  |
| *- 14 wks* | 0.70±0.02 | 0.69±0.03 | 0.74±0.02 | 0.70±0.01 |
| *- 16 wks* | 0.74±0.02 | 0.77±0.02 | 0.75±0.02 | 0.94±0.03***^,###^ |
| *- 18 wks* | 0.71±0.02 | 0.84±0.03 | 0.77±0.02 | 0.96±0.06** |
| *Wall thickness, systolic (mm)* |  |  |  |  |
| *- 14 wks* | 1.21±0.06 | 1.24±0.03 | 1.25±0.05 | 1.28±0.04 |
| *- 16 wks* | 1.14±0.06 | 1.26±0.03 | 1.32±0.03^#^ | 1.47±0.03*^,###^ |
| *- 18 wks* | 1.14±0.03 | 1.31±0.03 | 1.30±0.05 | 1.46±0.08 |
| End-diastolic volume, long-axis (μl) |  |  |  |  |
| *- 14 wks* | 53±3 | 53±2 | 47±3 | 46±3 |
| *- 16 wks* | 53±4 | 56±3 | 51±2 | 54±2 |
| *- 18 wks* | 55±2 | 64±3 | 49±5 | 58±3 |
| End-systolic volume, long-axis (μl) |  |  |  |  |
| *- 14 wks* | 21±3 | 21±2 | 16±2 | 17±2 |
| *- 16 wks* | 18±3 | 22±1 | 16±2 | 18±2 |
| *- 18 wks* | 21±3 | 26±2 | 16±2 | 23±2* |
| Ejection fraction (%) |  |  |  |  |
| *- 14 wks* | 60±5 | 62±2 | 66±2 | 62±2 |
| *- 16 wks* | 66±3 | 60±2 | 69±3 | 67±3 |
| *- 18 wks* | 63±3 | 60±2 | 69±3 | 61±3 |
| *Fractional shortening (%)* |  |  |  |  |
| *- 14 wks* | 34.0±3.5 | 29.8±2.0 | 33.9±1.9 | 32.5±1.3 |
| *- 16 wks* | 29.5±2.2 | 31.9±1.7 | 38.9±1.3^##^ | 35.3±1.6 |
| *- 18 wks* | 28.0±2.1 | 27.1±0.7 | 37.6±2.4^#^ | 28.6±3.3* |
| Stroke volume (μl) |  |  |  |  |
| *- 14 wks* | 31±2 | 33±1 | 31±2 | 28±2 |
| *- 16 wks* | 34±2 | 33±2 | 35±2 | 36±2 |
| *- 18 wks* | 34±2 | 38±2 | 33±3 | 36±3 |
| Cardiac output (ml.min^-1^) |  |  |  |  |
| *- 14 wks* | 12.0±1.1 | 12.6±0.6 | 11.9±0.7 | 10.4±0.8 |
| *- 16 wks* | 13.8±1.0 | 13.3±0.8 | 14.1±0.7 | 15.6±1.1 |
| *- 18 wks* | 12.4±1.1 | 14.7±1.4 | 12.8±1.3 | 15.1±1.1 |
| *E/A ratio* |  |  |  |  |
| *- 14 wks* | 1.79±0.21 | 1.58±0.10 | 1.95±0.13 | 1.76±0.10 |
| *- 16 wks* | 1.74±0.15 | 1.66±0.20 | 1.80±0.07 | 1.62±0.32 |
| *- 18 wks* | 1.69±0.17 | 1.75±0.13 | 2.10±0.14 | 1.76±0.20 |

Data are expressed as means ± S.E.M before treatment (14 wks) and after 2 (16 wks) and 4 weeks (18 wks) of treatment. * refers to effect of angiotensin II in non-diabetic and diabetic mice (* P < 0.05, ** P < 0.01, *** P < 0.001) and ^#^ refers to effect of diabetes in vehicle-treated and Angiotensin II-treated mice (# P < 0.05, ## P < 0.01, ### P < 0.001).
